# Prognosis of patients with heart disease with acute kidney injury undergoing dialysis treatment

**DOI:** 10.1590/0034-7167-2022-0022

**Published:** 2022-10-03

**Authors:** Daniela Ferreira, Maria Aparecida Batistão Gonçalves, Dayana Souza Fram, João Luiz Grandi, Dulce Aparecida Barbosa

**Affiliations:** I Universidade Federal de São Paulo. São Paulo, São Paulo, Brazil; II Instituto do Coração. São Paulo, São Paulo, Brazil

**Keywords:** Acute Kidney Injury, Patients, Heart Diseases, Prognosis, Dialysis., Lesión Renal Aguda, Pacientes, Cardiopatías, Pronóstico, Diálisis., Injúria Renal Aguda, Pacientes, Cardiopatias, Prognóstico, Diálise.

## Abstract

**Objectives:**

to verify the relationship of cardiovascular diseases with acute kidney injury and assess the prognosis of patients in renal replacement therapy.

**Methods:**

a cohort study, carried out in a public hospital specialized in cardiology. Treatment, comorbidities, duration of treatment, laboratory tests, discharge and deaths were analyzed.

**Results:**

of the 101 patients, 75 (74.3%) received non-dialysis treatment. The most frequent cardiological diagnoses were hypertension, cardiomyopathies and coronary syndrome. Hospitalization in patients undergoing dialysis was 18 days, hemoglobin <10.5g/dl and anuria in the first days of hospitalization contributed to the type of treatment. Each increase in hemoglobin units from the first day of hospitalization decreases the chance of dialysis by 19.2%. There was no difference in mortality.

**Conclusions:**

the main cardiological diseases were not predictive of dialysis indication, and clinical treatment was the most frequent. Anuria and anemia were predictors for dialysis treatment.

## RESUMO

**Objetivos:**

verificar a relação de patologias cardíacas com injuria renal aguda e avaliar o prognóstico do paciente em terapia de substituição renal.

**Métodos:**

estudo de coorte, realizado em hospital público especializado em cardiologia. O tratamento, comorbidades, tempo de tratamento, exames laboratoriais, alta e óbitos foram analisados.

**Resultados:**

dos 101 pacientes, 75 (74,3%) receberam tratamento não dialítico. Os diagnósticos cardiológicos mais frequentes foram hipertensão arterial, miocardiopatias e síndrome coronariana. A internação nos pacientes dialíticos foi de 18 dias, a hemoglobina <10,5g/dl e a anuria nos primeiros dias de internação contribuíram para o tipo de tratamento. Cada aumento de unidade de hemoglobina a partir do primeiro dia de internação diminui em 19,2% a chance de diálise. Não houve diferença na mortalidade.

**Conclusões:**

as principais doenças cardiológicas não foram preditivas de indicação de diálise, e o tratamento clínico foi o mais frequente. Anuria e anemia foram preditores para o tratamento dialítico.

## RESUMEN

**Objetivos:**

verificar la relación de las patologías cardíacas con la insuficiencia renal aguda y evaluar el pronóstico del paciente en terapia de reemplazo renal.

**Métodos:**

estudio de cohorte, realizado en un hospital público especializado en cardiología. Se analizó el tratamiento, las comorbilidades, la duración del tratamiento, los exámenes de laboratorio, el alta y las muertes.

**Resultados:**

de los 101 pacientes, 75 (74,3%) recibieron tratamiento no dialítico. Los diagnósticos cardiológicos más frecuentes fueron hipertensión arterial, miocardiopatías y síndrome coronario. La hospitalización en pacientes en diálisis fue de 18 días, la hemoglobina <10,5 g/dl y la anuria en los primeros días de hospitalización contribuyeron al tipo de tratamiento. Cada aumento de unidades de hemoglobina desde el primer día de hospitalización disminuye la posibilidad de diálisis en un 19,2%. No hubo diferencia en la mortalidad.

**Conclusiones:**

las principales enfermedades cardiológicas no fueron predictoras de indicación de diálisis, siendo el tratamiento clínico el más frecuente. La anuria y la anemia fueron predictores para el tratamiento de diálisis.

## INTRODUCTION

Acute kidney injury (AKI) is a very common and severe syndrome among clinical syndromes in Intensive Care Units (ICU)^(1-2)^. It presents a very complex clinical condition associated with high rates of mortality, morbidity and high health costs^(3-4)^. AKI is conceptualized as a syndrome characterized by abrupt loss of renal function (seven days or less), i.e., increased plasma urea and creatinine concentrations, usually accompanied by reduced urinary output (oliguria) and strongly associated with increased short- and long-term patient morbidity and mortality, and subsequent development of chronic kidney disease (CKD)^(1,5)^.

According to the Brazilian Society of Nephrology (2020), the main risk factors for AKI in Brazil are advanced age, diabetes mellitus (DM), diabetic nephropathy, cardiovascular diseases (heart failure, coronary disease, peripheral vascular disease) and hypertension^(6)^. In a Brazilian study, the incidence of AKI in critically ill patients was 40.5%, and the AKI dialysis rate was 13%. In this study, hypertension (p <0.017) and elevated serum creatinine concentration (p <0.001)^(7)^ were also highlighted as predictors of AKI on ICU admission. Another study showed an incidence of 44.7% of AKI development in patients admitted to ICU^(8)^.

Studies carried out in developing countries contribute to the recognition of the epidemiological overview of AKI^(1)^. However, research carried out with western populations showed that AKI represents a clinical syndrome with an incidence close to that of acute myocardial infarction (AMI)^(9)^, being associated with other factors, such as increased in-hospital mortality, prolonged hospital stay, and increased hospital costs^(3,7)^. The magnitude of risk factors is highly dependent on the presence of comorbidities, including cardiovascular diagnosis, hypertension, diabetes mellitus, and preexisting CKD^(10)^.

AKI is a multifactorial entity. The use of nephrotoxic drugs and hypovolemia were the main causes. Currently, other etiological factors contribute to its development, including iatrogenic events, such as arterial hypotension during surgeries, severe diarrhea secondary to fungal infection, especially by Clostridium difficile, use of nephrotoxic antibiotics (amphotericin B, aminoglycosides), non-steroidal anti-inflammatory drugs (NSAIDs), infections, sepsis, use of contrast agents for diagnostic procedures (e.g., coronary angiography, tomography, magnetic resonance imaging), cardiovascular diseases (mainly HF, AMI), advanced age, male, geographic location and socioeconomic status, which even considerably modify epidemiological data and future events of the disease^(11)^.

Cardiovascular diseases are still a cause of high mortality in ICUs around the world, as they are more frequently identified in patients with acute injury who have or have not undergone renal replacement therapy (RRT). Directly or indirectly, AKI has both acute and long-term adverse cardiovascular effects^(12)^.

Among other cardiovascular diseases that are strongly associated with AKI, cardiomyopathies are myocardial diseases with dysfunction, classifying them as dilated cardiomyopathy, hypertrophic, restrictive cardiomyopathy and arrhythmogenic right ventricular cardiomyopathy. There are numerous cases of patients with volume overload due to ischemic dilated cardiomyopathy developing AKI, and due to this situation, these patients required RRT while awaiting heart transplantation^(13)^.

Considering the above, we are motivated to carry out this study, which aims to verify the relationship between the main cardiovascular diseases and the occurrence of AKI and assess the prognosis of patients undergoing RRT.

## OBJECTIVES

To verify the relationship of the main cardiovascular diseases with the occurrence of AKI and assess the prognosis of patients submitted to RRT in a reference hospital in cardiology.

## METHODS

### Ethical aspects

This study was approved by the Research Ethics Committee of the *Universidade Federal de São Paulo* (UNIFESP).

### Study design, period, and location

This is a prospective study with a cohort design, using the instrument STROBE according to Equator guidelines, carried out from 2015 to 2018, with an active search for AKI cases in patients admitted to the clinical ICU of a public hospital specializing in cardiology, located in the central region of the city of São Paulo.

### Population/sample

The sample was for convenience and all patients who met the criteria were included in the study. The number of 101 patients was considered adequate for statistical analysis. The main search diagnoses were acute coronary syndrome (ACS), accounting for 25% of cases, exacerbation of acute heart failure (HF), with 15% of cases, valvular heart disease, with 10% of the global total. Non-cardiovascular primary diagnoses accounted for 50% of admissions and were marked by AKI, sepsis or acute respiratory failure (ARF).

### Inclusion and exclusion criteria

Patients over 18 years old, with serum creatinine level greater than 1.5 mg/dl, according to the classification corresponding to KDIGO^(5)^ (increase ≥ 50% of baseline serum creatinine),were included. Patients who underwent transplantation and had CKD were excluded.

### Data collection and study protocol

Patient identification data (age, sex), length and type of treatment, comorbidities, cardiological diagnoses of ICU admission, use of vasoactive drugs, antibiotics, diuretics and iodinated contrasts, dialysis treatment or not were collected. Dialysis treatment was defined as “use of RRT”^(14)^. Non-dialysis treatment was defined as correction of hydroelectrolytic and acid-base disorders, suspension of nephrotoxic drugs, correction of blood volume with hydration or association of loop diuretics and use of vasoactive drugs, in addition to patients’ outcome (categorized as discharged or dead), results of laboratory tests of urea, creatinine, potassium, sodium, hematocrit and hemoglobin in the first three days of hospitalization in the emergency room and the first three days of ICU hospitalization. Data collection was performed daily by the researcher, using Electronic Patient Records (EPR).

### Statistical analysis

Initially, spreadsheets were created in Microsoft Excel, where the data were inserted. The classification variables were presented in tables containing absolute (n) and relative (%) frequencies. The association of these variables with groups (dialytic/non-dialytic) was assessed with Pearson’s chi-square test or Fisher’s exact test. Kolmogorov-Smirnov test was used for assessing normality in the distribution of quantitative variables. Quantitative variables were presented descriptively in tables containing mean and standard deviation. The variable length of ICU stay was described as median and with interquartile limits. The profile of means of measures performed in the three days during ICU stay was assessed with analysis of variance for repeated measures. In all tests, p<0.05 was assigned to indicate statistical significance, and the significant values were marked with an asterisk (*).

## RESULTS

Of the 101 patients included, 75 (74.3%) received non-dialytic treatment and 26 (25.7%) underwent RRT, dialytic treatment. Patient sociodemographic characteristics are described in Table 1.

**Table 1:** Distribution of sociodemographic characteristics (sex and age) of patients undergoing dialytic and non-dialytic treatment, São Paulo, São Paulo, Brazil, 2021 (N=101)

Variables	Dialytic		Non-dialytic		Total		*p* value
	No.	%	No.	%	No.	%	
Male	18	69.2	38	50.7	56	55.0	0.101
Female	8	30.7	37	49.3	45	45.0	
Total	26	25.7	75	74.3	101	100.0	
Age ^**^ (years)	61		65				0.339
Standard deviation	14		16				

* *Pearson’s chi-square test;*

** Student’s t-test.

Regarding patients’ sex, a normal distribution between men and women is observed. However, when we analyzed sex separately in relation to dialytic treatment, it was observed that RRT was more used among men (18/69.2%) than among women (8/30.7%). No statistically significant differences were observed in terms of treatment and mean age between women and men (Table 1).

Table 2 shows the comorbidities and the main therapies used in patients with cardiological diagnosis.

**Table 2:** Distribution of comorbidities and main therapeutic methods performed in cardiological patients undergoing dialytic and non-dialytic treatment, São Paulo, São Paulo, Brazil, 2021 (N=101)

Variables	Dialytic		Non-dialytic		Total		*p* value
	No.	%	No.	%	No.	%	
Observed comorbidities							
Acute coronary syndrome	11	42.3%	37	49.3%	48	47.5	0.536 ^1^
Myocardiopathy	12	46.2%	32	42.7%	44	43.5	0.757 ^1^
Heart failure	12	46.2%	32	42.7%	44	43.5	0.017 ^1^
Myocardial revascularization	2	7.7%	16	21.3%	18	17.8	0.146 ^2^
Hypertension	13	50.0%	47	62.7%	60	59.4	0.257 ^1^
Diabetes mellitus	7	26.9%	26	34.7%	33	32.6	0.468 ^1^
Dyslipidemia	5	19.2%	26	34.7%	31	30.6	0.141 ^1^
Sepsis	9	34.6%	15	20.0%	24	23.7	0.131 ^1^
Chagas disease	3	11.5%	9	12.0%	12	11.8	1.000 ^2^
Obesity	3	11.5%	11	14.7%	14	13.8	1.000 ^2^
Smoking	4	15.4%	9	12.0%	13	12.8	0.736 ^2^
Main therapeutic methods observed							
Use of vasoactive drugs	22	84.6%	56	74.7%	78	77.2	0.297 ^1^
Use of antibiotics	20	76.9%	53	72.6%	73	72.2	0.667 ^1^
Use of diuretics	19	73.1%	46	61.3%	65	64.3	0.281 ^1^
Stent	7	26.9%	28	37.3%	35	34.6	0.336 ^1^
Use of contrast	9	34.6%	22	29.3%	31	30.6	0.615 ^1^
Use of renal protector	7	87.5%	17	73.9%	24	23.7	0.642^2^
Post-contrast hydration	4	57.1%	17	77.3%	21	20.7	0.357 ^2^

1 *Pearson’s chi-square test; ^2^Fischer’s exact test.*

Table 2 describes the cardiological diagnoses observed in patients admitted to the ICU. In patients undergoing dialysis, ACS was observed, with 11 (42.3%), and cardiomyopathies (dilated, hypertrophic and restrictive), with 12 (46.2%) of the records. Regarding metabolic comorbidities, hypertension had 50% of cases, and DM and dyslipidemia (DLP), with 26.8% and 19.2%. In the non-dialytic group, DM and DLP contributed with 34.7% of clinical diagnoses, while obesity was observed in 14.7% of cases. No significant differences were observed regarding the diagnostic methods used in the two groups.

Table 3 presents the results of the serum levels of the main renal markers (urea, creatinine, sodium and potassium), in addition to hemoglobin (Hb) and hematocrit (Ht) levels, collected from patients during the first three days of observation in the emergency room, which preceded ICU admission.

**Table 3:** Distribution of serum levels of laboratory tests (urea, creatinine, potassium, sodium, hemoglobin and hematocrit), verified on days 1, 2 and 3 of observation in the emergency room, São Paulo, São Paulo, Brazil, 2021 (N=101)

Variables	Dialytic			Non-dialytic			*p* value
	No.	Mean	SD	No.	Mean	SD	t-student
	25	34	16	72	39	17	0. 160
Urea - Day 1	26	104	76	75	77	36	0. 093
Urea - Day 2	14	97	75	27	73	37	0. 271
Urea - Day 3	12	102	80	17	69	40	0. 203
Creatinine - Day 1	26	2.29	1.55	75	1.78	0.71	0. 120
Creatinine - Day 2	14	2.47	2.23	27	1.66	0.93	0. 212
Creatinine - Day 3	12	2.72	2.74	17	1.52	0.87	0. 167
Potassium - Day 1	26	4.88	1.19	75	4.55	0.95	0. 156
Potassium - Day 2	14	4.05	0.60	27	4.26	0.82	0. 411
Potassium - Day 2	12	4.23	0.98	17	3.94	0.57	0. 325
Sodium - Day 1	26	1.36	8	75	1.38	5	0. 408
Sodium - Day 2	14	1.36	6	27	1.39	6	0. 127
Sodium - Day 3	12	1.36	5	17	1.40	6	0. 068
Hemoglobin - Day 1	24	11.0	3.10	73	12.80	2.59	0.006
Hemoglobin - Day 2	14	-10.96	1.67	27	11.79	2.10	0.003
Hemoglobin - Day 3	12	-10.30	1.76	17	12.06	2.44	0.003
Hematocrit - Day 1	24	34.2	23	73	17.7	7.1	0.035
Hematocrit - Day 2	14	30.3	11	27	4.0	5.9	0.002
Hematocrit - Day 3	12	17.9	5	17	6.6	7.1	0.003

*SD - standard deviation.*

**Table 4:** Distribution of clinical variables (anuria), length of stay in the Intensive Care Unit and outcome (discharge or death) of patients undergoing dialytic and non-dialytic treatment, São Paulo, São Paulo, Brazil, 2021 (N=101)

Variables	Dialytic	Non-dialytic	Não dialíticos		Total		*p* value
	No.	%	No.	%	No.	%	
Anuria ^1^							0. 002^*^
Yes	9	34.6	6	8.0	15	14.9	
No	17	65.4	69	92.0	86	85.1	
Total	26	25.7	75	74.3	101	100.0	
Clinical outcome							0. 123
Discharge	13	50.0	28	32.9	41	40.6	
Death	13	50.0	47	67.1	60	59.4	
Total	26	25.7	75	74.3	101	100.0	
Length of stay in the ICU (days)	26		75				0. 001^*^
Median	18		10				
Interquartile variation	(10-31)		(7-21)				0. 014

*Pearson’s chi-square test - degrees of freedom 0.5;*

1 Anuria - Fischer’s exact test.

Regarding the renal markers (urea and creatinine), the results in the three samples on consecutive days were not statistically significant. However, when we observe Hb and Ht results, there was a decrease in values, with statistically significant differences in the second (p= 0.003 and 0.002) and third day (p=0.003), respectively.

The results demonstrate that the presence of anuria was more frequent in the dialytic group (34.2%) than in the non-dialytic group (8%), with *p*= 0.002. Regarding the length of hospital stay, the median was statistically significant (*p*=0.01) in dialytic patients (18 days), compared to non-dialytic patients (10 days) (Table 3).

Regarding deaths, 50% occurred in the dialytic group and 67.1% in the non-dialytic group. The risk of death in the dialytic group was 1.46 (95% CI) times greater than in the non-dialytic group.

As we can see in patients with present diuresis, the chance of dialysis is higher when Hb is less than 10.5 g/dl. In patients with anuria, the Odds Ratio increases the chance of being subjected to RRT by 6,694 times.

**Figure 1:**
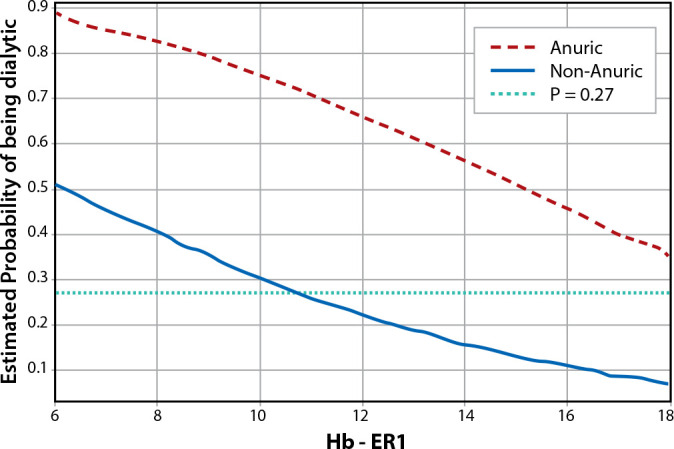
Logistic regression chart for anuria and hemoglobin on the first day in the emergency room for dialytic and non-dialytic patients

## DISCUSSION

The present study analyzed the data of 101 patients; of these, 75 (74.5%) underwent non-dialytic treatment, and 26 (25.7%) underwent RRT. In their study, Cardoso *et al.* ^(15)^ analyzed 109 patients. Of these, 19 (52.6%) underwent RRT treatment, and 90 (27.1%) underwent non-dialytic treatment. In another study^(16)^, reporting 103 patients, the authors observed a rate of 38.8% of patients requiring dialysis. In this study, conducted with 1,300 patients, the authors found an AKI frequency of 37.2%, of which 15.5% evidenced the need for dialysis^(17)^. Importantly, there are few studies on AKI in the ICU that compare groups of patients undergoing RRT and the group of patients undergoing non-dialytic treatment.

Regarding sex, in the present study, there was a predominance of males 18 (69.2%) in the dialytic group and 38 (50.7%) in the non-dialytic group. In a study with 41 adult patients with AKI, they were 67 [54 - 77] years old. Of these, 28 (68.3%) were male^(18)^. In the national and international literature, other studies are found that corroborate with AKI diagnosis, being more frequent in men (60%^(19)^, 68.3%^(18)^ and 54.8%^(20)^).

Regarding age, the median was 61 years in the dialytic group and 65 years in the non-dialytic group, with a standard deviation of 14 years in the dialytic group and 16 years in the non-dialytic group, comparable data with two other studies^(18-21)^. According to the results of this study, that relate age with a risk factor for AKI, no agreement was found in the literature regarding the association of age as a risk factor for AKI, especially when associated with preexisting chronic diseases in older adults. However, it is observed that in recent decades, worldwide, AKI has a higher prevalence in aging populations^(21)^.

In the present study, in the two groups analyzed (dialytic and non-dialytic), the same cardiovascular diseases were observed. Hypertension, ACS and cardiomyopathies were the comorbidities present in most patients, who had a higher probability of a less favorable prognosis, regardless of the association with AKI.

Of the non-cardiological clinical diagnoses associated with AKI that presented the highest frequency, in the dialytic group, DM, with 26.9%, and DLP, with 19.2%. In the non-dialytic group, DM and DLP had the same frequency (34.7%), and obesity contributed with 14.7% of cases. Non-cardiovascular diseases have become more prevalent and may contribute to morbidity and mortality. The results of DM in the present study support those found in other studies^(8,22)^. In the presence of atherosclerotic events, AKI was not associated^(23)^.

In a study on risk factors for AKI in the ICU, it was observed that 7.5% of patients had hypertension^(2,22)^, HF, hypovolemia, use of vasoactive drugs, such as noradrenaline, dobutamine, dopamine and use of antibiotics^(2,11,24)^. It was concluded that having three or more associated risk factors increased the possibility of developing the disease^(2)^.

Of the cardiological diagnoses observed in patients, the main ones were myocardial revascularization, ACS, AMI with and without elevation, cardiomyopathies, hypertension and Chagas disease. The analysis of associations of cardiologic comorbidities and AKI in the literature is scarce. In the present study, the main cardiovascular diseases observed in the group of patients on dialytic treatment were diagnoses of hypertension (50.0%), cardiomyopathies (46%), and ACS (42.3%). In the non-dialytic group, there was a predominance of hypertension (62.7%), ACS (49.3%) and cardiomyopathies (dilated, hypertrophic and restrictive), with 42.7% of all cases.

Among the cardiovascular risk factors, some studies have highlighted hypertension^(2,23)^ and HF with statistical significance, being considered the main factors associated with the development of AKI^(25-26)^. Another study points to AMI as one of the most prevalent diagnoses among cardiovascular diseases (25.9%)^(12,27)^. In a more recent study, it was observed that AKI is associated with excessive risks of death^(28)^, CKD progression and cardiovascular events, although previous studies have important limitations^(12,23)^.

Supported by other studies^(25,29)^, hospital mortality in our population was high, with 50% and 67.1% in dialytic and non-dialytic patients, respectively. The relative risk of death in the dialytic group was 1.5 times higher compared to the non-dialytic group. Another study demonstrates that AKI is associated with excessive risks of death, CKD progression and cardiovascular events^(23)^.

### Study limitations

The relatively low number of patients made it impossible to assess the results in a multivariate model, and the observational study was carried out in a single center specialized in cardiology dependent on usual practice of a single service. Future studies with a broader sample and that support the relevance of comparability between the two groups are encouraged.

### Contributions to health

On the other hand, the importance of the finding on low Hb as a predictor of AKI and dialytic treatment and a higher risk of death can guide the conduct in the early correction of this condition. Thus, it is necessary to continue this study, aiming at reducing morbidity and mortality, which affects the population of patients undergoing dialytic treatment.

## CONCLUSIONS

Of the cardiological comorbidities associated with AKI in both groups, hypertension, cardiomyopathies and ACS were more frequent. The anuria factor increases by seven times the possibility of a patient undergoing dialytic treatment.

It was identified that, for each increase of one unit of Hb, the chance of a patient requiring RRT decreased by 19.2%. In the logistic regression, we verified that, in the presence of anuria and low Hb, the chance of dialysis is higher. The risk of death was 1.46 times higher in the dialytic group than in the non-dialytic group.

## References

[1] Santos RP, Carvalho ARS, Peres LAB. (2019). Incidence and risk factors of acute kidney injury in critically ill patients from a single centre in Brazil: a retrospective cohort analysis. Sci Rep.

[2] Benichel CR, Meneguin S. (2020). Fatores de risco para lesão renal aguda em pacientes clínicos intensivos. Acta Paul Enferm.

[3] Zuk A, Palevsky PM, Fried L, Harrel FE, Khan S, Mckay DB (2018). Overcoming translational barriers in acute kidney injury: a report from an NIDDK workshop. CJASN.

[4] Korula S, Balakrishnan S, Sundar S, Paul V, Balagopal A. (2016). Acute kidney injury-incidence, prognostic factors, and outcome of patients in an Intensive Care Unit in a tertiary center: a prospective observational study. Indian J Crit Care Med.

[5] KDIGO AKI Work Group (2012). Kidney Int. Suppl. KDIGO clinical practice guideline for acute kidney injury [Internet].

[6] Sociedade Brasileira de Nefrologia (2021). Insuficiência Renal[Internet].

[7] Zampieri FG, Soares M, Borges LP, Salluh JIF, Ranzani OT. (2017). The Epimed Monitor ICU Database®: a cloud based national registry for adult intensive care unit patients in Brazil. Rev Bras Ter Intensiva.

[8] Guedes JR, Silva ES, Carvalho ILN, Oliveira MD. (2017). Incidência e fatores predisponentes de insuficiência renal aguda em unidade de terapia intensiva. Cogitare Enferm.

[9] Rossaint J, Zarbock A. (2016). Acute kidney injury: definition, diagnosis and epidemiology. Minerva Urol Nefrol [Internet].

[10] Fortrie G, Geus HRH, Betjes MGH. (2019). The aftermath of acute kidney injury: a narrative review of long-term mortality and renal function. Crit Care.

[11] Ferreira D, Bragança AC, Volpini RA, Shimizu MHM, Gois PHF, Girardi ACC (2019). Vitamin D deficiency is a potential risk factor for lipid Amphotericin B nephrotoxicity. PLoS Negl Trop Dis.

[12] Holland EM, Moss TJ. (2017). Acute Noncardiovascular Illness in the Cardiac Intensive Care Unit. JACC.

[13] Vallabhajosyula S, Dunlay SM, Murphree DH, Barsness GW, Sandhu GS, Lerman A (2019). Cardiogenic Shock in Takotsubo Cardiomyopathy Versus Acute Myocardial Infarction: an 8-year national perspective on clinical characteristics, management, and outcomes. JACC Heart Fail.

[14] Kellum JA, Ronco C. (2016). The 17th Acute Disease Quality Initiative International Consensus Conference: introducing precision renal replacement. Blood Purif.

[15] Cardoso BG, Carneiro TA, Magro MCS. (2017). Recuperação de pacientes com lesão renal aguda dialítica e não dialítica. Cogit Enferm [Internet].

[16] Nascimento GVR, Silva MN, Carvalho JD, Feitosa LR, Antão JD. (2020). Outcomes in acute kidney injury in noncritically ill patients lately referred to nephrologist in a developing country: a comparison of AKIN and KDIGO criteria. BMC Nephrol.

[17] Malhotra R, Kashani KB, Macedo E, Kim J, Bouchard J, Wynn S (2017). A risk prediction score for acute kidney injury in the intensive care unit. Nephrol Dial Transplant.

[18] Gaião SM, Gomes AA, Paiva JAOC. (2016). Prognostics factors for mortality and renal recovery in critically ill patients with acute kidney injury and renal replacement therapy. Rev Bras Ter Intensiva.

[19] Kellum JA, Romagnani P, Ashuntantang G, Ronco C, Zarbock A, Anders HJ. (2021). Acute kidney injury. Nat Rev Dis Primers.

[20] Silva ABV, Cavalcante AMRZ, Taniguchi FP. (2018). Survival and risk factors among dialytic acute kidney injury patients after cardiovascular surgery. Braz J Cardiovasc Surg.

[21] Hoste EAJ, Kellum JA, Selby NM, Zarbock A, Palevsky PM, Bagshaw SM (2018). Global epidemiology and outcomes of acute kidney injury. Nat Rev Nephrol.

[22] Lopes D, Schran LS, Oliveira JLC, Oliveira RBSR, Fernandes LM. (2018). Fatores de risco/causais para insuficiência renal aguda em adultos internados em terapia intensiva. Enferm Brasil.

[23] Ikizler TA, Parikh CR, Himmelfarb J, Chinchilli VM, Liu KD, Coca SG (2021). A prospective cohort study of acute kidney injury and kidney outcomes, cardiovascular events, and death. Kidney Int.

[24] Vasco CF, Watanabe M, Fonseca CD, Vattimo MFF. (2018). Sepsis-induced acute kidney injury: kidney protection effects by antioxidants. Rev Bras Enferm.

[25] Rigonatto MCL, Magro MCS. (2018). Risk for acute kidney injury in primary health care. Rev Bras Enferm.

[26] Moura ELB, Amorim FF, Huang W, Maia MO. (2017). Contrast-induced acute kidney injury: the importance of diagnostic criteria for establishing prevalence and prognosis in the intensive care unit. Rev Bras Ter Intensiva.

[27] Horne KL, Packington R, Monaghan J, Reilly T, Selby NM. (2017). Three-year outcomes after acute kidney injury: results of a prospective parallel group cohort study. BMJ Open.

[28] Liao Z (2020). Study on Related Factors of Acute Kidney Injury in Patients under Intensive Care. Proceed Anticancer Res.

[29] Heung M, Yessayan L. (2017). Renal replacement therapy in acute kidney injury: controversies and consensus. Crit Care Clin.

